# Local Adaptation at the Transcriptome Level in Brown Trout: Evidence from Early Life History Temperature Genomic Reaction Norms

**DOI:** 10.1371/journal.pone.0085171

**Published:** 2014-01-14

**Authors:** Kristian Meier, Michael Møller Hansen, Eric Normandeau, Karen-Lise D. Mensberg, Jane Frydenberg, Peter Foged Larsen, Dorte Bekkevold, Louis Bernatchez

**Affiliations:** 1 National Institute of Aquatic Resources, Technical University of Denmark, Silkeborg, Denmark; 2 Department of Bioscience, Aarhus University, Aarhus C, Denmark; 3 Département de Biologie, Institut de Biologie Intégrative et des Systèmes (IBIS), Pavillon Charles-Eugène-Marchand, Université Laval, Québec, Canada; University of Arkansas, United States of America

## Abstract

Local adaptation and its underlying molecular basis has long been a key focus in evolutionary biology. There has recently been increased interest in the evolutionary role of plasticity and the molecular mechanisms underlying local adaptation. Using transcriptome analysis, we assessed differences in gene expression profiles for three brown trout (*Salmo trutta*) populations, one resident and two anadromous, experiencing different temperature regimes in the wild. The study was based on an F2 generation raised in a common garden setting. A previous study of the F1 generation revealed different reaction norms and significantly higher Q_ST_ than F_ST_ among populations for two early life-history traits. In the present study we investigated if genomic reaction norm patterns were also present at the transcriptome level. Eggs from the three populations were incubated at two temperatures (5 and 8 degrees C) representing conditions encountered in the local environments. Global gene expression for fry at the stage of first feeding was analysed using a 32k cDNA microarray. The results revealed differences in gene expression between populations and temperatures and population × temperature interactions, the latter indicating locally adapted reaction norms. Moreover, the reaction norms paralleled those observed previously at early life-history traits. We identified 90 cDNA clones among the genes with an interaction effect that were differently expressed between the ecologically divergent populations. These included genes involved in immune- and stress response. We observed less plasticity in the resident as compared to the anadromous populations, possibly reflecting that the degree of environmental heterogeneity encountered by individuals throughout their life cycle will select for variable level of phenotypic plasticity at the transcriptome level. Our study demonstrates the usefulness of transcriptome approaches to identify genes with different temperature reaction norms. The responses observed suggest that populations may vary in their susceptibility to climate change.

## Introduction

Environmental heterogeneity is one of the most important factors influencing evolutionary trajectories of species and populations. If different populations inhabit environments that are spatially heterogeneous, whereas at the same time the environments are predictable and show limited temporal heterogeneity, then this is expected to promote diversifying selection, potentially leading to local adaptation [1]. If, on the other hand, environments are unpredictable and fluctuate temporally, then the range of phenotypes that can be expressed from the same genotype, i.e. phenotypic plasticity. becomes important [2]. Hence, local adaptation and phenotypic plasticity can be viewed as opposite responses to environmental heterogeneity in space and time, although the level of plasticity may also be subject to local selection [3].

Local adaptation can be tested according to a “home *versus* away” criterion, where the fitness of populations in native and non-native environments is compared, or a “foreign *versus* local” criterion, where fitness of local and non-local populations is compared in the local environment of one of the populations [1]. In practice, local adaptation can be tested according to these criteria using reciprocal transplant experiments, either in a natural setting or more indirectly in an experimental setting where populations are tested under different environmental conditions mimicking those encountered in their natural environments. In the latter case, reaction norms could be analysed, and crossing reaction norms among populations would suggest differences in genotype-by-environment interactions, and thereby local adaptation in these populations [4]. Moreover, analyzing reaction norms provides information about phenotypic plasticity within and among populations [4], [5].

Salmonids are renowned for their natal homing, which restricts gene flow between populations and may favor adaptation to local environments. Several studies have suggested that salmonids are locally adapted [6]–[8]. A recent review concluded that local adaptation is present in ca. 55–70% of interpopulation comparisons in salmonids [7]. Conversely, this also implies that not all populations are expected to be locally adapted. The review also found local adaptation to be generally more pronounced at larger geographical scales but, depending on the selection regimes and the underlying functional loci in question, it may occur at smaller scales of just a few kilometres [7].

The advent of the genomic era has drastically improved our capacity to analyze both neutral and potentially functional gene-linked markers [9]–[11]. Combined with hitch-hiking mapping approaches [12]–[14], this allows for the identification of markers, chromosome regions, and in some cases even traits that are potentially under selection [15], [16]. Furthermore, analysis of variation in gene expression at the transcriptome level offers a powerful tool to assess the role of gene regulation in adaptive divergence [17]–[19] and adaptive plasticity [20] among natural populations. Several of these tools and approaches have been applied to study local adaptation and adaptive plasticity in salmonids at different levels of organization, including DNA, the transcriptome, and phenotypic traits [7], [21]–[29].

Gene expression varies among tissues, environments and developmental stages, which makes it more complex to analyze than DNA sequence variation. As a result, gene expression is best treated as a phenotype of the studied organism [30]. However, this complexity offers the possibility of obtaining insight into the genes and biological processes underlying physiological adaptations [31]–[34], for example by studying different life stages or assessing gene expression in different environments. Analysis of gene expression has also revealed that differences in transcription profiles can be used in a predictive framework, for instance in predicting individual premature death in sockeye salmon (*Oncorhynchus nerka*) returning to spawn [35]. Similarly, transcriptome studies of resident and anadromous salmonid populations identified candidate genes and physiological pathways involved in preparatory adaptations to these different life histories [31], [32], [36]. Analysis of one of these candidate genes in brown trout revealed an expression signal months before migration, which holds potential for predicting individuals’ future life history tactics [37]. As shown by these examples, transcriptome studies hold great promise for identifying biomarkers that could be used for monitoring and forecasting important developments in populations, such as emerging stress responses due to pathogens or high temperature [37], [38].

Once local adaptation has been inferred at higher level phenotypic traits, at the morphological or life history level, it becomes particularly interesting to study the underlying patterns at the transcriptome level. A recent study of early life history traits in brown trout (*Salmo trutta*) revealed divergent temperature adaptation among populations on a small geographical scale (< 40 km) for length in alevins and at the stage of first feeding [39]. These traits had significantly higher Q_ST_ [40] values compared to F_ST_, suggesting diversifying selection. This study was based on four natural populations, for each of which an F1 generation was reared in a common garden setting. The offspring were reared at three temperatures representing the conditions experienced from egg incubation until fry emergence in their respective rivers. The authors found significant population level differences in reaction norms for several early life history traits. Temperature reaction norms for length at the alevin and first feeding stages revealed that the populations had a higher growth at the temperature experienced in their home environment compared to the foreign environment, suggesting local adaptation.

The present study builds upon the findings of Jensen *et al.* [39]. We applied transcriptomic tools to identify the genes and possible physiological pathways underlying the temperature-related adaptations suggested by this study. In order to analyse temperature genomic reaction norms at the transcriptomic level, we used fish from three of the four populations studied by Jensen *et al*. [39]. Specifically, we used fry at the stage of first feeding in the F2 generation and reared them at two different temperatures. We used a ∼32,000 cDNA microarray developed for salmonids by the consortium for Genomic Research on All Salmon Project (cGRASP) [41], [42]. Patterns of gene expression from whole fry were analysed in order to test for temperature adaptation at the transcriptome level by identifying genes showing temperature-related plasticity. Among genes displaying temperature-related plasticity, focus was directed towards those that were expressed differently between ecologically different populations in order to identify genes involved in thermal adaptation. Since the studied populations are genetically close (F_ST_  =  0.060 based on ten microsatellite loci; [39]), transcription profiles were expected to show only small differences. We also expected that within-population plasticity could be linked to environmental predictability, such as resident trout encountering a more uniform environment during their life cycle as compared to anadromous trout.

## Materials and Methods

### Studied populations

**Figure 1:**
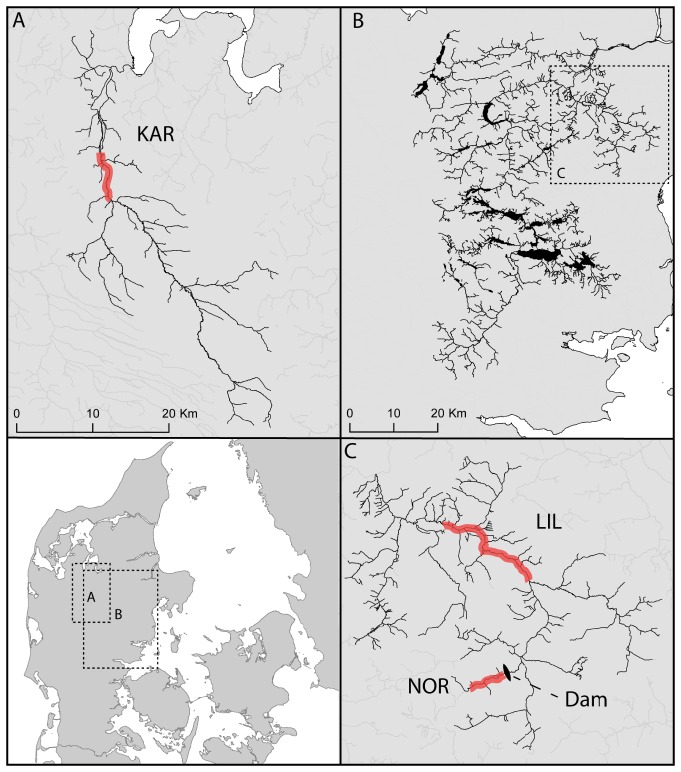
Location of populations. Map of Denmark showing the location of the rivers inhabited by the sampled populations. a) Karup River (KAR) which runs into the Limfjord region. b) The Gudenaa River system. c) Lilleaa (LIL) and Norring Moellebaek River (NOR), which runs into the Gudenaa River system. The population inhabiting NOR is resident; immigration has been blocked by a dam established in the 15^th^ century. The sections of the rivers where the original parent fish were caught are indicated by red.

Brown trout populations from three rivers were studied: the Karup (KAR), Norring Moellebaek (NOR) and Lilleaa Rivers (LIL). These rivers experience different temperature regimes during the period lasting from egg incubation until fry emergence (see supporting information for Jensen *et al.* [39]). KAR runs into the Limfjord region (Fig. 1) and is inhabited by anadromous brown trout. Temperature in the river system is affected both by ambient temperature and groundwater, the latter of which usually elevates water temperatures during winter to ca. 6–8°C [39]. As a result, temperature is expected to vary throughout the river system. NOR runs into LIL, which in turn runs into the Gudenaa River system (Fig. 1). However, gene flow between the two populations is restricted by a dam, built ca. 1500. Thus, NOR is inhabited by land-locked resident brown trout that have been isolated for ca. 500 years corresponding to ca. 143 generations assuming a generation length of 3.5 years [43], whereas LIL is inhabited mainly by anadromous brown trout. The isolation of NOR is reflected in a F_ST_ value of 0.062 between LIL and NOR, whereas F_ST_ between the two anadromous populations LIL and KAR is lower, 0.023 (based on ten microsatellite loci; [39]). Both rivers are influenced by ambient air temperature and as a result the winter (i.e. at early life stages) temperature in these two river systems is lower (typically 3–5°C) (Table 1).

**Table 1:** Studied brown trout populations.

		Winter water temperature	Life history strategy
Population	abbr.		
Lilleaa River	LIL	Low (3–5°C)	Anadromous
Karup River	KAR	Varying (3–8°C)	Anadromous
NorringMoellebaek River	NOR	Low (3–5°C)	Resident

Population names and abbreviation (abbr.), winter water temperature, and life history strategy. LIL and NOR are fed by run-off water and as a result the temperature during winter is low (typically 3–5°C; see Fig. S1 in [39]). In KAR the temperature is expected to vary in the river since it is affected by run-off and ground-feed water.

### Rearing experiment

During the autumn of 2004 and the winter of 2004/2005, adults from the three populations were collected by electro fishing and brought to the Danish Centre for Wild Salmon. Electrofishing was conducted by technical staff at the Danish Institute for Fisheries Research, who had all necessary permits from the Danish Ministry of Food, Agriculture and Fisheries and followed all required regulations. The fish were stripped for milt and eggs to establish an F1 generation [39]. In autumn/winter 2008/2009, mature F1s were used to establish F2 offspring for each of the three populations. For KAR and LIL, 20 full-sib families nested within 5 half-sib families were established, whereas for NOR, 15 full-sib families nested within 5 half-sib families were established. Eggs from each full sib family were divided into two pools that were incubated at 5 and 8°C in separate hatching troughs. Coolers ensured that the temperature did not deviate by more than ± 0.5 degrees. As all females were not ripe at the same time, crosses were conducted at different dates. However, fertilized eggs were incubated in the same hatching troughs experiencing identical temperatures and other conditions throughout the experiment. Incubation and rearing of fish followed standard hatchery procedures requiring no animal ethics permit.

Jensen *et al.* [39] found evidence for local adaptation in early life history traits in both newly hatched alevins and fry at the stage of first feeding. We chose to focus on the latter stage since we expected more genes to be detectably transcribed with progression in development [44]. Also, this is the life stage in nature at which the fry would emerge from the gravel in the spawning redds and become exposed to predation, other sources of mortality and thereby natural selection; mortality is expected to be as high as 90% during the first year of life [45]. At the time of first feeding (ca. 750–780 day-degrees), 10 individuals from each family and temperature were collected and transferred to separate tubes containing RNAlater (QIAGEN, Hilden, Germany). The stage of first feeding was evaluated based on the elapsed time in terms of day-degrees along with visual inspection of the absorption of the yolk sac, conducted by experienced staff at the Danish Centre of Wild Salmon. Following storage at 4°C overnight, the RNAlater in the tubes was changed and the samples were then stored at –20°C until RNA extraction.

### Microarray analysis

The 32K cDNA microarray developed for salmonids by cGRASP was used for the analyses [41], [42]. Twenty individuals representing 10 families (thus representing two replicates for each family) were randomly selected from each population and each temperature. RNA was extracted from whole individuals on ice using the RNA Pure Link Minikit (Invitrogen, Paisley, UK). Each fry was homogenized individually in 0.8ml lysis buffer containing 1% 2-mercaptoethanol using a Tissuelyzer (QIAGEN, Hilden, Germany). Samples were then centrifuged at 2600 g for 5 min and 0.6 ml of the supernatant was transferred to a new 2 ml tube, 0.6 ml Trizol and 0.6 ml chloroform was added, mixed and centrifuged at 13,000 g at 4°C for 15 min. Then, 800 µl of the upper phase was transferred to a new tube and RNA was extracted according to the manufacturer’s recommendations. After RNA extraction, 1 µl of RNase inhibitor (20 units/µl, Ambion, Invitrogen, Paisley, UK) was added to prevent RNA degradation and samples were DNase treated with DNase I, amplification grade (1 unit/µl) (Invitrogen, Paisley, UK) following the manufacturer’s recommendation. RNA quantity and integrity was checked on a Nanodrop instrument (ND-1000; Thermo Fisher Scientific Inc., Wilmington, Delaware, USA) and on an Experion automated electrophoresis station using RNA HighSens chips (BIORAD, Copenhagen, Denmark). Following quality control, 15 µg of RNA was reverse transcribed, labeled and hybridized following the protocol that can be found at: http://web.uvic.ca/grasp/microarray/array.html. In short, 15 µg mRNA was reverse transcribed using superscript II retrotranscriptase (Invitrogen, Paisley, UK) and Cy3/Cy5 dyes (Genisphere, Hatfield, Pennsylvania, USA). Microarrays were prepared by washing two times in 0.1% SDS for five minutes and five times in milli-Q water for 1 minute and dried by spinning. The cDNA was hybridized to the arrays with formamide buffer, competitor DNA (LNA dt blocker) and human cot DNA in hybridization chambers placed in water baths at 51°C for 16 hours. The arrays were then washed at 51°C for five minutes in 2×SSC/0.1% SDS, twice in 1×SSC, twice in 0.1×SSC and dried by centrifugation. The Cy5/Cy3 fluorescent dyes (3DNA capture reagent, Genisphere, Hatfield, Pennsylvania, USA) were hybridized to the attached cDNA with formamide buffer and human cot DNA in hybridization chambers at 51°C for two hours. The microarray washing and drying procedure was repeated as above.

Microarrays were scanned using a ScanArray scanner (Perkin-Elmer Life Sciences, Waltham, MA, USA). Spots were localized and quantified with the QuantArray 3.0 software (Perkin- Elmer Life Sciences), using the histogram quantification method and keeping the mean value of intensity for each spot. Local background was removed and the data from bad spots were manually excluded from the data set. For each of the two dyes on each array, spots with signal intensities lower than the mean intensity of the empty spots plus twice their standard deviation were flagged as non-expressed. Spots that had no non-expressed flag in at least one of the six studied groups (3 rivers and 2 temperatures) were kept. This left a total of 8588 spots for the analysis that were common for all the groups. After a base two logarithm transformation, the data were normalized according to the rlowess method (regional lowess procedure) implemented in R/MAANOVA package [46] to remove signal intensity-dependent and region-dependent dye effects on each slide.

### RT-qPCR analysis

Real time polymerase chain reaction (RT-qPCR) was used to confirm the microarray results for a subset of the data. To cross validate results, we extracted RNA from novel individuals from a subset of the families analysed with the microarrays. We tested for a population effect between LIL and NOR at 5°C and for a temperature effect for LIL. For each population, 12 individuals representing 6 families were analysed. RNA was extracted as for the microarray experiment, but without the trizol/chloroform purification step since less RNA was needed for the RT-qPCR. Afterwards, RNA quantity was measured on a Nanodrop (ND-1000) instrument. We applied the TURBO DNA-free™ kit (Applied Biosystems, Carlsbad, California, USA) with removal agent according to the manufacturer’s instructions to ensure DNA-free samples for RT-qPCR. Afterwards, oneµl of RNase inhibitor (20 unit/µl, Ambion) was added to prevent RNA degradation. RNA integrity was verified on a 2% agarose gel. Finally, oneµg of RNA was reversed transcribed using the High-Capacity cDNA Reverse Transcription Kit (Applied Biosystems) according to the manufacturer’s instructions. The cDNA was diluted 20 times before use. Primers were designed for the following genes, considered representative examples for temperature and population effects: Gluthathione S-transferase P (Genbank accession nr: EG845649): GSTP-F: 5′-AGCCGACTACGCCCTGGTGC-3′, GSTP-R: 5′-GGGTCGGGCACACAGCCTCT-3′, product size: 112 bp. This gene was tested for a temperature effect; Ependymin precursor (Genbank accession nr: CB502684): EPD-F: 5′-ACCACCAGGCATGAACGGGC-3′, EPD-R: 5′-CGCTCCTGCAGAAGGGCTGC-3′, product size: 116bp. This gene was tested for a population effect. Reference genes were selected among the 8588 genes, based on low fold changes (FC) among groups and small standard deviations within group in the microarray results. The following genes were selected: Mitochondrial 28S ribosomal protein S32 (Genbank accession nr: CB510473),RPS32-F: 5′-TCCTCGATGGTGGGGGCCTG-3′, RPS32-R: 5′-AGACCCCCTGGCCAATCCGG-3′, product size: 99bp;Glyceraldehyde-3-phosphate dehydrogenase (Genbank accession nr: CA047823), GAPDH-F: 5′-GGGGGCGAAAGGACTTCCAGG-3′, GAPDH-R: 5′-ACCCCAAGGTGAAATGGAGCACC-3′, product size: 80 bp. PCR products from all primer pairs were electrophoresed on a 2% agarose gel to verify primer specificity and PCR product length. Removal of genomic DNA was also verified in this step. RT-qPCR was performed on the Ligthcycler480 (Roche Diagnostics, Mannheim, Germany) using SYBR GreenI Master (Roche Diagnostics) and the Lightcycler Relative Quantification Software 3.5 (Roche Diagnostics). The PCR reactions were performed as follows: 95°C for 10 min, followed by 40 cycles of 95°C for 20 s, 60°C for 20 s and 72°C for 10 s. Finally, melting curve analysis was performed to control for the presence of more than one product in the reaction. Negative control reactions without any template as well as dilution series were conducted to control for contamination and the efficiency of primer pairs, respectively.

### Statistical analysis

To test for gene expression differences among populations, temperature levels and interactions between populations and temperatures, based on the microarray results, we used the MANOVA/R package [46]. Data was corrected for intensity linked bias using a R-LOWESS package and analysed with an ANOVA test under a mixed model with dye, population, temperature and population × temperature as fixed factors and array and family as random factors. A permutation based F test (1000 permutations) was applied to detect significant differences. To determine the degree of plasticity in the populations, each population was analysed separately for a temperature effect. We applied an ANOVA test under a mixed model to each population with temperature as fixed effect and array and family as random factors. A permutation based F test (1000 permutations) was applied to detect significant differences. All results were corrected with the False Discovery Rate (FDR) [47].

For statistical analysis of RT-qPCR results, we used the Relative Expression Software Tool (REST) 2009 software [48]. The software determines whether target genes are differently expressed and is able to use several reference genes for normalization by taking the geometric mean of these into account. The significance of the determined ratios is tested by bootstrapping and randomization methods.

### Gene Ontology analysis

We performed a gene ontology (GO) analysis using the Blast2GO V.2.5.0 software [49], [50]. Briefly, the software conducts 1) BLAST [51], 2) mapping and 3) annotation. A BLAST search first identifies homologs to the input sequence in databases of annotated sequences. GO annotation is then transferred from the homologs to the queried sequences. After the annotation transfer, we used Blast2GO to conduct an enrichment analysis using Fisher’s exact test to find over represented GO terms. We applied this test to compare our groups of significant genes with either a population effect, temperature effect or an interaction effect tested against all the cDNA clones analysed.

## Results

### Microarray analysis

Of the 32,000 cDNA clones spotted on the array, 8588 were transcribed above noise level in samples from the three analysed brown trout populations. The two-way ANOVA identified population, temperature and interaction effects after FDR correction.

### Statistical analysis

**Figure 2:**
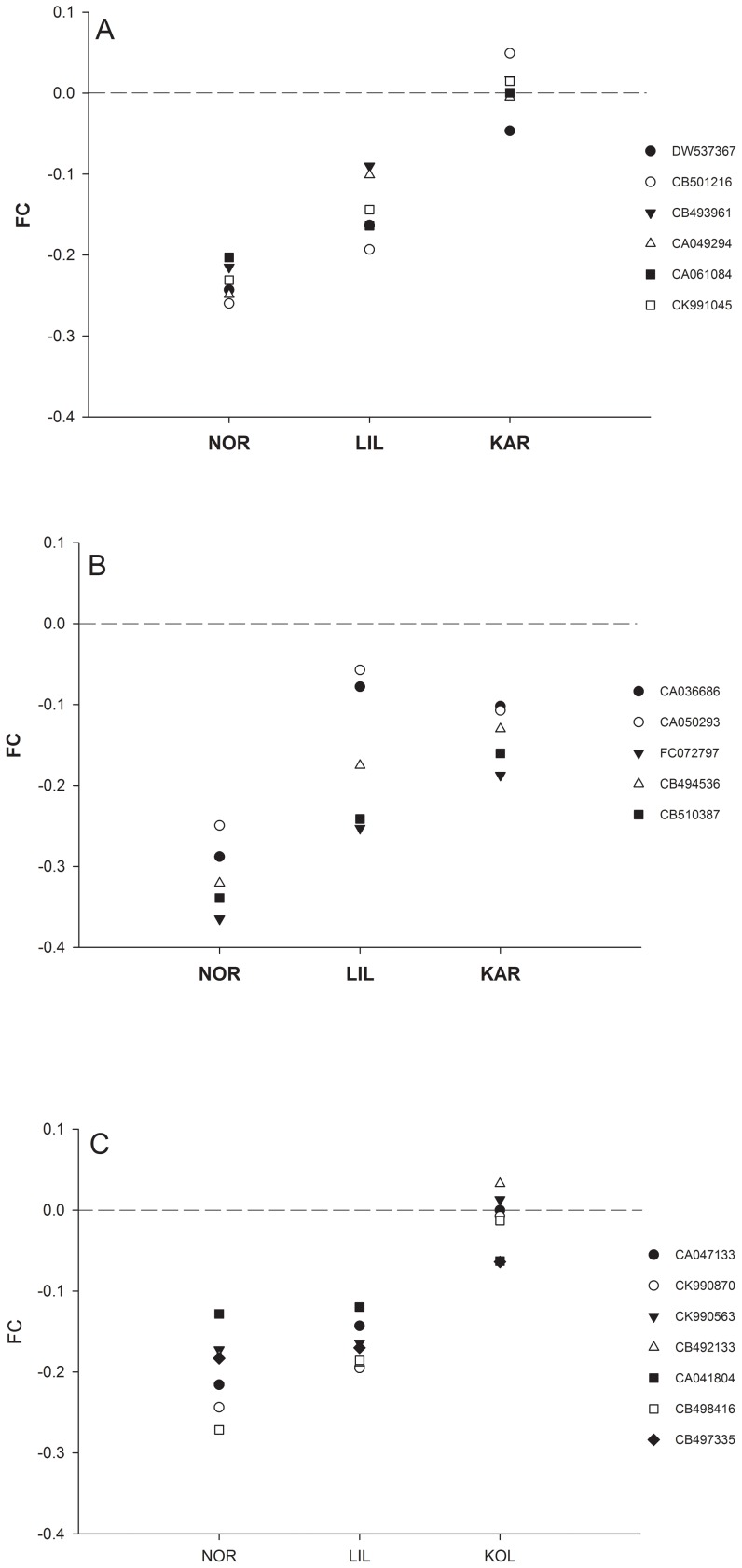
Expression of Hemoglobin genes. Base 2 logarithms of the fold changes between the two temperatures for Hemoglobin genes with a significant population effect. a) Hemoglobin subunit alpha, b) Hemoglobin subunit beta, c) Hemoglobin subunit beta 1. A positive fold change indicates a higher level of expression at 5°C. The codes refer to Genbank accession numbers.

For the population effect, 174 cDNA clones (2% of the clones) were significantly differently expressed between the three populations (*p* < 0.05 after FDR correction). Large differences in FC were revealed (FC: 0.18–1.57). Among these, 22 represented hemoglobin subunits that were differently expressed with FC in the range of 0.56–1.22. These included the following hemoglobin subunits: Hemoglobin subunit alpha, alpha 1, alpha-4, beta, beta-1 and beta 4, with alpha, beta and beta-1 being represented by more than two cDNA clones. The pattern of transcription differed between these subunits. For hemoglobin subunit alpha and beta-1, expression was up-regulated at higher temperatures for LIL and NOR whereas small differences were found for KAR between the two temperatures (Fig. 2). For hemoglobin beta, all populations exhibited up-regulated expression at higher temperatures (Fig. 2).

**Figure 3:**
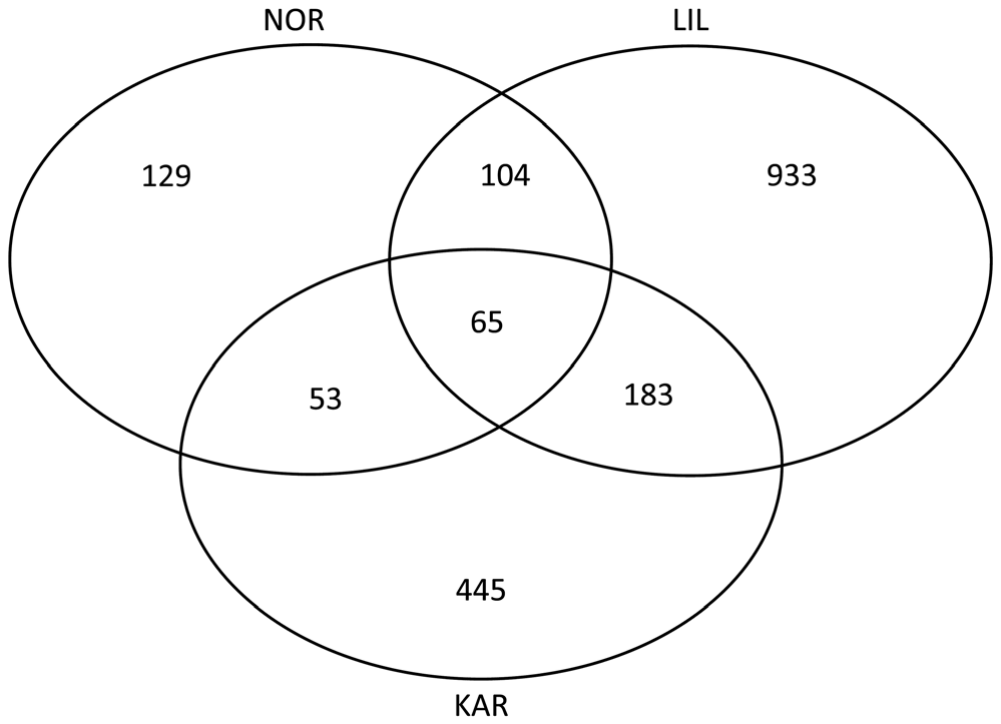
Gene expression differences among populations. Venn diagram showing differently expressed genes between the two temperatures in the three populations: NOR, LIL, and KAR. Numbers represent the number of differently expressed cDNA clones among the two temperatures for each population as well as the number of differently expressed cDNA clones shared among the populations.

Compared to the population effect, temperature effects were low, with FC ranging from -0.54 to 0.45. The 2454 cDNA clones that were differently expressed between temperatures (28.6%) revealed different levels of plasticity for the studied populations. More specifically, subsequent analysis of the temperature effect for each population taken separately revealed differences in the number of cDNA clones being differently expressed (Fig. 3). LIL had the largest plasticity with 1285 clones being differently expressed between the two temperatures followed by KAR with 746 cDNA clones and NOR with 351 cDNA clones (χ^2^  =  597.3, 2 d.f., p < 0.0001).

**Figure 4:**
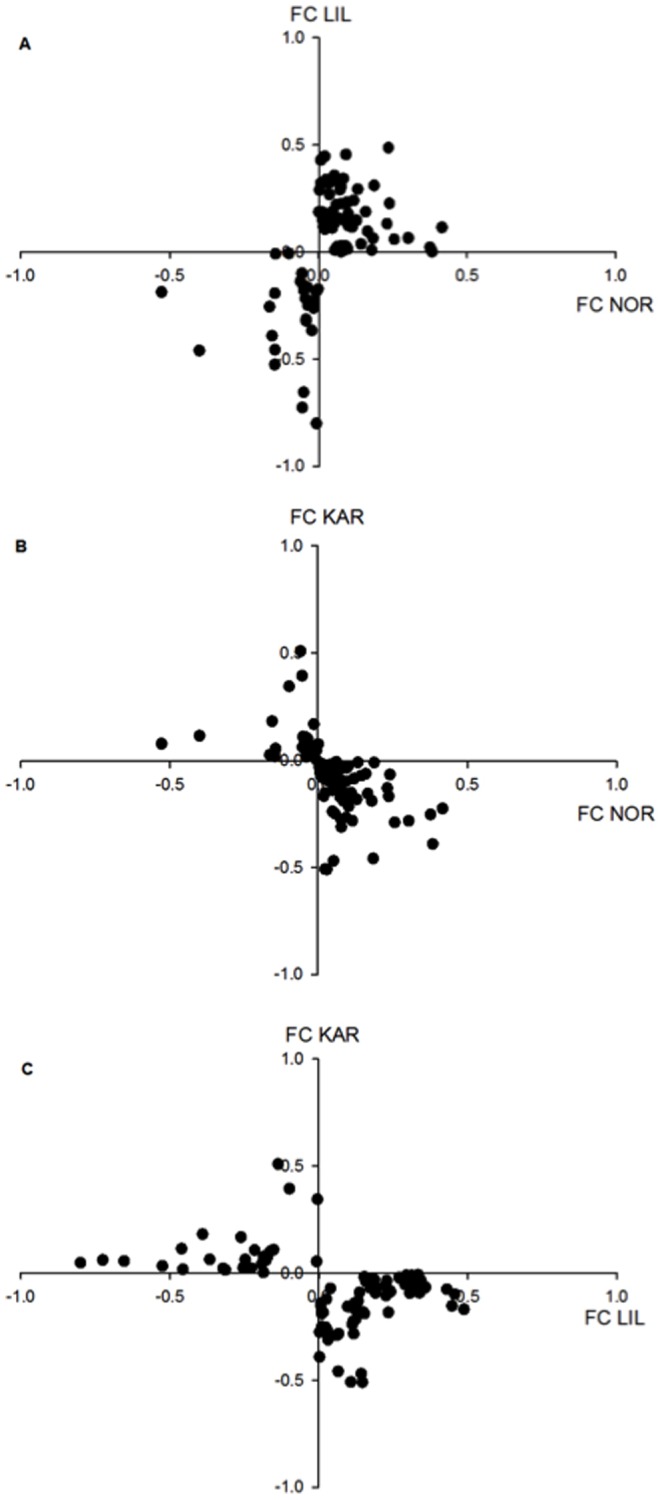
Changes in gene expression between temperatures. Vector plot of base 2 logarithm of fold changes in gene expression between the two temperatures for each population. Each axis represents the fold change for one population. A positive value indicates a higher level of expression at 5°C. Only genes with an interaction effect that are differently expressed between KAR compared to NOR and LIL are plotted. a) Fold changes for NOR plotted against fold changes for LIL. b) Fold changes for NOR plotted against fold changes for KAR. c) Fold changes for LIL plotted against fold changes for KAR.

**Figure 5:**
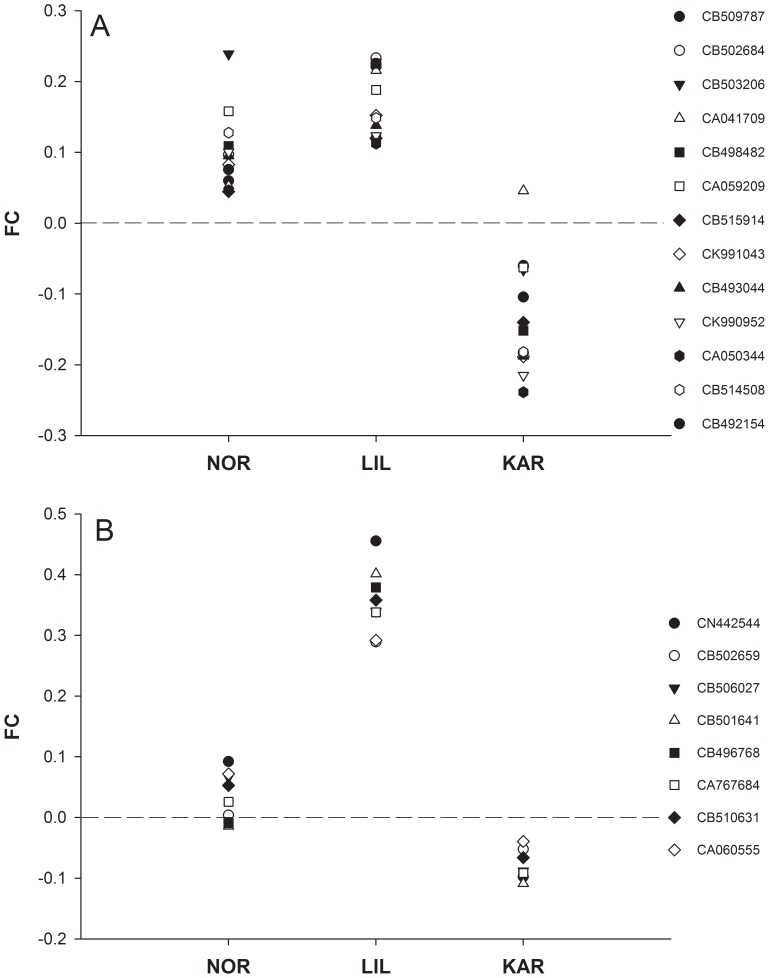
Changes in gene expression between temperatures Ependymin precursor and H-2 class II histocompatibility antigen genes. Base 2 logarithm fold changes between the two temperatures for Ependymin precursor and H-2 class II histocompatibility antigen gamma chain genes with a significant interaction effect. a) Ependymin precursor and ependymin precursor 1, b) H-2 class II histocompatibility antigen gamma chain genes. A positive fold change indicates a higher level of expression at 5°C. The codes refer to Genbank accession numbers.

A total of 385 cDNA clones (4.5%) had a significant interaction effect with FC in the range of 0.13–0.99 (*p* < 0.05 after FDR correction). Based on the work of Jensen *et al.* [39] temperature reaction norms vary among these three populations with NOR and LIL experiencing low temperatures during incubation whereas for KAR temperature conditions vary throughout the river. We therefore focused on genes that were differently expressed between KAR and both LIL and NOR (Fig. 4). Of the 385 cDNA clones with an interaction effect, 90 were differently expressed between KAR compared to NOR and LIL (Fig. 4). Among these, 63 were up-regulated at higher temperature in KAR and down-regulated in NOR and LIL (Table 2). Twenty-seven were down-regulated at higher temperature in KAR and up-regulated in LIL and NOR (Table 3). In terms of specific genes, Ependymin precursors and H-2 class II histocompatibility antigen gamma chain had a significant interaction effect for several cDNA clones with a consistent pattern of expression. Thirteen Ependymin precursor cDNA clones had a significant interaction effect (Fig. 5.a). Twelve of these revealed different transcription between KAR and both NOR and LIL (Table 2). Eight H-2 class II histocompatibility antigen gamma chaincDNA clones showed a significant interaction effect (Figure 5.b), among which six showed different transcription between KAR compared to the two other populations (Table 2).

**Table 2:** Gene expression reaction norms between populations.

Interaction effect		Fold change Temperature				
FDR corrected p-value	Fold change	NOR	LIL	KAR	Accession number	Description
0.038	0.233	0.187	0.311	–0.010	CB491393	40S ribosomal protein S13
0.033	0.139	0.067	0.157	–0.037	CB496534	40S ribosomal protein S14
0.036	0.238	0.143	0.039	–0.071	CB498077	40S ribosomal protein S14
0.010	0.325	0.165	0.096	–0.155	CB498290	Acidic mammalian chitinase precursor
0.003	0.710	0.416	0.115	–0.224	CB496513	Acidic mammalian chitinase precursor
0.000	0.631	0.302	0.066	–0.282	CA039104	AF275939 Salvelinus fontinalis progastricsin mRNA, complete cds
0.004	0.210	0.016	0.119	–0.167	CB506272	AF358667 Oncorhynchus mykiss procathepsin B mRNA, complete cds
0.025	0.306	0.055	0.010	–0.249	CK991342	AF363273_1 toxin-1 [Oncorhynchus mykiss]
0.000	0.542	0.028	0.146	–0.509	CB497670	Cathepsin L precursor
0.000	0.534	0.021	0.107	–0.507	CK990496	Cathepsin L precursor
0.001	0.519	0.050	0.142	–0.470	CB502503	Cathepsin L precursor
0.028	0.187	0.062	0.025	–0.121	CB494645	CD59 glycoprotein precursor
0.003	0.720	0.375	0.023	–0.253	CB509772	Chymosin precursor
0.009	0.213	0.132	0.293	–0.010	EG855211	Claudin-4
0.037	0.185	0.095	0.228	–0.035	CB497097	Coagulation factor XIII B chain precursor
0.020	0.253	0.072	0.009	–0.172	CK990929	Coiled-coil domain-containing protein 28A
0.026	0.173	0.052	0.172	–0.068	CB506394	Cytochrome b5
0.007	0.279	0.097	0.015	–0.183	CB497393	Cytochrome c oxidase subunit 5B, mitochondrial precursor
0.026	0.652	0.183	0.065	–0.458	CA045170	Elastase-2A precursor
0.009	0.373	0.230	0.132	–0.130	CK990664	Embryonic pepsinogen precursor
0.010	0.199	0.060	0.221	–0.060	CB509787	Ependymin precursor
0.000	0.311	0.096	0.233	–0.183	CB502684	Ependymin precursor
0.010	0.307	0.239	0.227	–0.066	CB503206	Ependymin precursor
0.004	0.260	0.109	0.119	–0.152	CB498482	Ependymin precursor
0.004	0.221	0.158	0.188	–0.063	CA059209	Ependymin precursor
0.047	0.199	0.045	0.120	–0.140	CB515914	Ependymin-1 precursor
0.007	0.283	0.083	0.152	–0.189	CK991043	Ependymin-1 precursor
0.014	0.275	0.095	0.138	–0.188	CB493044	Ependymin-1 precursor
0.002	0.316	0.100	0.124	–0.215	CK990952	Ependymin-1 precursor
0.009	0.296	0.046	0.112	–0.239	CA050344	Ependymin-1 precursor
0.026	0.310	0.128	0.149	–0.182	CB514508	Ependymin-2 precursor
0.007	0.234	0.076	0.226	–0.104	CB492154	Ependymin-2 precursor
0.021	0.191	0.001	0.187	–0.030	FC072796	Ferritin, heavy subunit
0.013	0.149	0.099	0.179	–0.026	CA051332	Ferritin, heavy subunit
0.000	0.578	0.255	0.060	–0.289	CB498673	Gastricsin precursor
0.018	0.405	0.179	0.010	–0.189	CB498165	Gastricsin precursor
0.033	0.430	0.008	0.429	–0.075	BU965890	Glyceraldehyde-3-phosphate dehydrogenase
0.013	0.410	0.092	0.456	–0.098	CN442544	H-2 class II histocompatibility antigen gamma chain
0.007	0.291	0.004	0.289	–0.052	CB502659	H-2 class II histocompatibility antigen gamma chain
0.035	0.315	0.066	0.340	–0.088	CB506027	H-2 class II histocompatibility antigen gamma chain
0.005	0.335	0.026	0.338	–0.092	CA767684	H-2 class II histocompatibility antigen gamma chain
0.008	0.328	0.053	0.358	–0.066	CB510631	H-2 class II histocompatibility antigen gamma chain
0.044	0.246	0.072	0.292	–0.039	CA060555	H-2 class II histocompatibility antigen gamma chain
0.010	0.197	0.014	0.187	–0.081	CB502631	H-2 class II histocompatibility antigen, I-E beta chain precursor
0.010	0.239	0.037	0.269	–0.021	CA058459	HLA class II histocompatibility antigen gamma chain
0.013	0.282	0.076	0.305	–0.093	CN442518	HLA class II histocompatibility antigen, DP alpha chain precursor
0.019	0.282	0.059	0.333	–0.007	CA042975	Intermediate filament protein ON3
0.000	0.476	0.236	0.487	–0.168	CB510438	Intestinal mucin-like protein
0.004	0.285	0.083	0.343	–0.035	CB496507	Intestinal mucin-like protein
0.033	0.238	0.118	0.241	–0.085	CN442510	NADH-ubiquinone oxidoreductase chain 1
0.017	0.459	0.021	0.447	–0.152	CK990278	Oncorhynchus mykiss invariant chain S25-7 mRNA, complete cds
0.003	0.860	0.383	0.002	–0.391	CB510981	Pepsin A precursor
0.004	0.389	0.076	0.031	–0.310	CB492550	Plasma retinol-binding protein 2
0.025	0.366	0.092	0.028	–0.267	CA053773	PREDICTED: similar to Palmd-prov protein [Danio rerio]
0.026	0.246	0.083	0.007	–0.150	CN442508	SAL319819 Salvelinus alpinus mitochondrial 12S rRNA gene
0.009	0.394	0.113	0.116	–0.282	FC072778	SMOEPDSSII Altantic salmon ependymin (SS-II) gene, complete cds
0.031	0.223	0.073	0.006	–0.139	CA055131	Solute carrier family 25 member 40
0.047	0.142	0.013	0.152	–0.017	CB517188	U3 small nucleolar ribonucleoprotein protein IMP3
0.046	0.300	0.034	0.319	–0.057	CK990488	UNKNOWN
0.043	0.165	0.054	0.136	–0.089	CA043079	UNKNOWN
0.003	0.324	0.007	0.321	–0.074	CA051952	UNKNOWN
0.036	0.359	0.075	0.002	–0.275	CB507633	UNKNOWN
0.035	0.200	0.048	0.190	–0.092	EG851313	UNKNOWN

Genes with a significant interaction effect that are up-regulated in KAR and down-regulated in NOR and LIL. A positive fold change indicates a higher level of expression at 5°C. P-values are corrected by the False Discovery Rate (FDR) procedure [47]. Fold changes are given in base 2 logarithms.

**Table 3:** Gene expression reaction norms between populations.

Interaction effect		Fold change Temperature				
FDR corrected p-value	Fold change	NOR	LIL	KAR	Accession number	Description
0.034	0.187	–0.037	–0.164	0.100	EG774444	Actin, cytoplasmic
0.000	0.680	–0.055	–0.725	0.062	CA054619	CTGF, CCN2, HCS24, IGFBP8: Connective tissue growth factor precursor
0.012	0.234	–0.036	–0.247	0.065	CB507314	Cytochrome c oxidase copper chaperone
0.045	0.242	–0.145	–0.008	0.055	CA057484	Deoxyuridine 5'-triphosphate nucleotidohydrolase, mitochondrial precursor
0.047	0.178	–0.008	–0.186	0.004	CK991244	Epiphycan precursor
0.013	0.192	–0.147	–0.192	0.043	CA063124	F-actin-capping protein subunit alpha-1
0.016	0.454	–0.099	–0.005	0.345	CB503169	Fish-egg lectin
0.046	0.582	–0.060	–0.137	0.510	CA061489	Fish-egg lectin
0.028	0.463	–0.056	–0.099	0.395	CB516765	Fish-egg lectin
0.037	0.354	–0.023	–0.366	0.065	CB493709	Fructose-bisphosphate aldolase A
0.004	0.412	–0.157	–0.390	0.182	EG769324	Glycogen phosphorylase, muscle form
0.005	0.278	–0.042	–0.314	0.016	CA055371	GTP-binding protein SAR1a
0.000	0.612	–0.050	–0.653	0.056	EG865212	Heat shock 70 kDa protein
0.003	0.692	–0.527	–0.186	0.077	CK990883	Hemoglobin subunit beta-2
0.000	0.347	–0.147	–0.456	0.018	CB492165	Histone H1x
0.020	0.169	–0.049	–0.177	0.062	CB510557	Interferon regulatory factor 2-binding protein 2-A
0.003	0.517	–0.400	–0.460	0.115	EG814010	L-lactate dehydrogenase A chain
0.025	0.307	–0.016	–0.261	0.168	DY711129	Myosin heavy chain, fast skeletal muscle
0.025	0.229	–0.044	–0.215	0.108	CB497762	Myosin light chain 1, skeletal muscle isoform
0.043	0.286	–0.042	–0.321	0.023	CB493766	Myosin light chain 3, skeletal muscle isoform
0.043	0.213	–0.017	–0.227	0.023	CB510226	Parvalbumin-2
0.001	0.419	–0.148	–0.525	0.034	CB493768	Triosephosphate isomerase
0.038	0.185	–0.002	–0.173	0.076	CB511010	Troponin T, cardiac muscle isoforms
0.020	0.193	–0.053	–0.152	0.110	EG787797	UNKNOWN
0.000	0.795	–0.007	–0.800	0.048	CA060788	UNKNOWN
0.049	0.206	–0.165	–0.254	0.025	CA061696	UNKNOWN
0.010	0.238	–0.016	–0.250	0.023	DY700362	UNKNOWN

Genes with a significant interaction effect that are down-regulated in KAR and up-regulated in NOR and LIL. A positive fold change indicates an increased expression from 8 to 5°C. P-values are corrected by the False Discovery Rate (FDR) procedure [47]. Fold changes are given in base 2 logarithms.

### RT-qPCR

Real time quantitative PCR confirmed the microarray results (Fig. S1): Gluthathione S-transferase P was significantly up-regulated in LIL at 5°C compared to 8°C (FC = 1.55, p<0.001) and Ependymin precursor was significantly up-regulated in LIL compared to NOR at 5°C (FC = 2.19, p = 0.012) after normalizing expression to the reference genes mitochondrial 28S ribosomal protein S32 andglyceraldehyde-3-phosphate dehydrogenase.

### Gene Ontology analysis

For the cDNA clones with a population effect, enrichment analysis identified 31 GO terms that were overrepresented, among which 11 represented biological processes. These processes included oxygen transport, immune system processes, and cell wall macromolecule catabolic processes (Table S1). For the enrichment analysis of the cDNA clones showing a temperature effect, 99 GO terms were overrepresented, 42 of which represented biological processes. These processes included oxygen and gas transport, the electron transport chain, respiration, sensory organ development, cold response, and the immune response, among others (Table S2). A total of 148 GO terms were overrepresented comparing cDNA clones with a temperature × population interaction effect involving all three populations. Biological processes represented 95 of these, which included several biosynthetic, catabolic and metabolic processes. Additionally, these included immune responses and regulation of muscle system processes (Table S3). Enrichment analysis of the 90 cDNA clones with a temperature x population interaction effect involving KAR vs. NOR and LIL resulted in 20 GO terms that were overrepresented, 10 of which represented biological processes. These included cell adhesion, immune response and intracellular protein transport (Table S4).

## Discussion

The aim of this study was to apply a transcriptomic approach to identify genes underlying temperature genomic reaction norms and temperature adaptation in brown trout. Based on the close genetic and geographic relationship among populations [39], we expected to detect few gene expression differences among populations. We also expected the degree of plasticity to vary according to each environment’s predictability in terms of temperature variations. By measuring gene expression levels in individuals from natural populations and raised in a common garden setting, we provided evidence for significant differences in gene expression among populations, temperatures, and their interaction. These findings of both plasticity and temperature × population interactions suggest local adaptation to temperature regimes in these populations. Most importantly, we identified several genes that showed different temperature reaction norms between the populations similar to the early life history reaction norm differences identified in a previous study of the same populations [39]. We discuss these results and their relevance in the following sections but first consider potential sources of error.

A number of factors could have biased the results. First, maternal effects can bias the observed phenotype. However, the analysed fry were F2 offspring raised in a common garden setting thereby minimizing this possible source of bias. The fact that these fish were raised in the lab for two generations could however introduce a bias from adaptation to captivity. Captivity over just a few generations can result in adaptations to the captive environment [52] and studies have revealed differences at the transcriptomic level after 5–7 generations of artificial selection [53]. Since our study is based on temperature adaptation for an early life history trait, we argue that adaptation to captivity should have little influence on the obtained results, although we cannot rule this out entirely. Moreover, since all F1 fish were reared under the same conditions, this if anything should decrease differences among populations.

It should be mentioned that some of the transcriptional differences observed could be caused by epigenetic modifications, such as DNA methylation or histone modification, rather than variation within regulatory sequences controlling gene expression. Such epigenetic changes can affect the accessibility of DNA and hence its transcription level [54]. Even though we cannot rule out these epigenetic factors, we note that by raising our populations in a common environment for two generations, modifications caused by the environment should be minimized between populations.

Finally, our study design is limited by the lack of replicates for each type of population studied (anadromous low temperature, anadromous variable temperature and resident low temperature). Whereas we can compare reaction norms at the levels of early life history traits [39] and transcriptomes (this study) for the same populations, the design does not allow for drawing general conclusions about gene expression in the different types of populations, although hypotheses can be proposed that could be tested in subsequent studies.

### Evidence for local adaptation

Several cDNA clones were significantly differently expressed among populations and temperature conditions. For most of these, an interaction effect with temperature was also observed. These two results taken together suggest the presence of local adaptation. Hemoglobin cDNA clones, however, were an exception to this scenario. Most of these cDNA clones varied in their expression level among the populations but showed no interaction with temperature. More precisely, these clones were up-regulated at higher temperatures for NOR and LIL whereas the pattern was more inconsistent at different temperatures for KAR (Fig. 2). It is thus possible that the lack of an interaction could be explained by the larger inter-individual variation found for KAR. Since oxygen affinity diminishes as temperature increases, [55] and that NOR and LIL are up-regulating hemoglobin transcription outside their normal temperature range, it could imply that hemoglobin expression has been selected to correspond to local environmental temperatures and has a plastic stress response outside the normal temperature range. This is supported by a recent microarray study of upper thermal tolerance in Artic charr (*Salvelinus alpinus*), which revealed that hemoglobin expression was down-regulated in heat tolerant *versus* intolerant fish [56]. More recently, strong parallel patterns of hemoglobin gene expression were observed between dwarf and normal whitefish (*Coregonus clupeaformis, Salmonidae*), indicating that these genes evolved under divergent selection in the predicted direction, that is higher hemoglobin gene expression in dwarf whitefish to accommodate higher energetic demand for active swimming [57]. In Atlantic salmon, the genomic organization of the hemoglobin genes has been characterized, revealing more copies of hemoglobin genes than found in other teleost species [58]. The authors propose that the higher number of copies could be a result of the tetraploid ancestry of salmonid fishes [59] combined with the diverse environments the Atlantic salmon has to deal with due to its complex life history. Given that the variety of hemoglobin subunits we identified as differently expressed among populations showed distinct expression patterns, our results support similar diversification of functions in brown trout, made possible by the ancient genome duplication in Salmonidae [59]. Similar results have been obtained in a completely different organism, *Daphnia pulex*, where extensive duplication of genes is found throughout the genome [60]. In this species, paralog gene copies show divergent patterns of expression under different environmental conditions, thus providing clear evidence that the paralogs have acquired different functions and are involved in responses to environmental variability.

The 90 cDNA clones that were found to be differently expressed between the ecologically different populations with respect to temperature (KAR compared to NOR and LIL) were involved in immune related responses, adhesion, and protein localization (Table S4). Ependymin, a secretory glycoprotein is known to play a role in adhesion and MHC class II genes in the immune response, as well as protein localization. Ependymin and MHC class II genes have previously been found to exhibit differences in gene expression levels between European flounder (*Platichthys flesus*) populations experiencing different salinities [33], as well as between wild and farmed strains of Atlantic salmon [53]. Studies of zebrafish (*Danio rerio*) and carp (*Cyprinus carpio*) have revealed that expression of the Ependymin gene is induced upon acclimation to cold [61]. We therefore propose that this gene contributes to a temperature-related stress/adaptive response.

Previous studies also revealed that expression of MHC class II genes varies with temperature in salmonids. In a study of rainbow trout (*Oncorhynchus mykiss*), fish were exposed to low temperatures (2°C), resulting in a down-regulation of MHC class II genes expression compared to fish kept at 12–13°C [62]. Temperature dependent expression of MHC class II genes was also found for brook charr (*Salvelinus fontinalis*) [63]. Furthermore the study revealed that a temperature sensitive minisatellite plays a role in the expression of the MHCIIβ (the gene encoding the β chain of MHC II) transcripts. In our study, we quantified global expression levels at 5 and 8°C. Despite such small temperature differences, we observed population specific temperature regulation. For NOR and LIL, expression levels of H-2 class II histocompatibility antigen gamma chain transcripts were higher at low temperatures, whereas they showed an opposite pattern in KAR. Based on these different temperature reaction norms, and the fact that NOR and LIL naturally experience these low temperatures in their natal rivers, this suggests that the temperature-related limit of immune responses could be shaped by selection allowing cold adapted populations to respond to pathogens at low temperatures.

In addition to Ependymin and MHC Class II, a number of other ecologically important genes showed significant genomic reaction norms (Tables 2 and 3). For instance, Ferritin is involved in both iron metabolism and immune responses and shows increased expression in response to bacterial infection [64]. Fish-egg lectins are assumed to be involved in innate immune responses of eggs and embryos [65], [66]. Cathepsins are lysosomal proteases [67]. They have been found to play a major role in breakdown of muscle protein during spawning migration in adult salmonids, thus providing energy for swimming and gonad development [68]. However, they are also assumed to play important roles in early life stages during yolk sac metabolism [67]. Finally, Heat Shock Proteins are involved in responses to temperature and other types of environmental stress, notably by acting as chaperones interacting with other proteins [69]. In total, the significant temperature x population interactions observed combined with the ecological importance of several of the genes are strong indicators of local adaptation involving multiple traits and processes.

### Plasticity within populations

Analysis of the temperature effect revealed a clear plastic response among the populations in response to temperature changes of only 3°C. This temperature difference caused small expression fold changes, although for several biological processes (Table S2). Analysis of the temperature effect for each population revealed a clear difference in the number of cDNA clones being differently expressed at the different temperatures. This degree of plasticity could be linked to different life history strategies linked to the predictability of the respective environments of these populations. More specifically, the resident NOR population had the lowest temperature-related plasticity of gene expression compared to the two anadromous KAR and LIL populations. These differences could be explained by the fact that the environment of the resident NOR population varies less compared to that of the two other populations, which experience different environments throughout their life cycle as they migrate from fresh- to saltwater. While our study design does not allow for an in-depth analysis of the role of environmental predictability on shaping plasticity, it nevertheless provides a rationale for using resident and anadromous populations to address that question.

### Evolutionary potential and future adaptation

The biological consequences of current climate change are accumulating rapidly [70] and there are increasing concerns about how species will respond or adapt to these changes [71]. There is growing evidence for measurable ecological [72] and evolutionary responses [73]–[75] in several species, for example changes in phenology and range shifts. It has been argued that the major part of genetic responses to climate change is expected to involve phenology, i.e. seasonal timing of important elements of life history, such as reproduction in order to track the optimal growing season [76]. In salmonids, this would primarily translate into shifting the spawning period. However, evolutionary change at traits of importance to physiological temperature response may also be important in salmonid fishes. For instance, the period from egg incubation until fry emerge from the gravel constitutes a critical phase of the salmonid life cycle. Eggs and larvae are unable to escape unfavorable temperature conditions and evolutionary adaptation may therefore occur. In the present study, we provided evidence for population-level differences in transcriptome profiles for fitness-related genes at the stage of first feeding, suggesting adaptation to local temperature conditions, in accordance with a recent Q_ST_ - F_ST_ study involving the same populations [39]. Yet, the findings of our transcriptome study also suggest local levels of plasticity for short-term adaptation (temperature effect) as well as temperature adaptation (interaction effect), the latter potentially involving trade-offs (for example metabolic costs [69]) under increased temperatures. Since the resident NOR population is adapted to low temperatures and demonstrates a reduced plasticity when compared to the other populations, it is probably the most vulnerable. At globally increasing temperatures, this local adaptation may prove to be maladaptive and the reduced plasticity may cause the populations to be negatively impacted by increasing environmental stochasticity, which is a predicted climate change scenario for the region (http://www.dmi.dk/dmi/index/klima/fremtidens_klima-2/ekstremt_vejr.htm).

## Conclusions

Our study demonstrates that temperature genomic reaction norms for several ecologically important genes are parallel to reaction norms observed for early life-history traits in the same populations [39]. Hence, the transcriptome data provide further evidence for local adaptation of the populations to local temperature regimes along with information on candidate genes and important physiological pathways. The finding of pronounced variation in plasticity among populations merits further consideration. Indeed, our study design is unable to resolve the specific mechanisms underlying this. For example, does it reflect differences in plasticity between anadromous and resident populations or is it due to other factors? It would be highly interesting in future studies to analyze more populations representing different environments and life histories to test the general hypothesis that the degree of environmental heterogeneity encountered by individuals throughout their life cycle determines the level of phenotypic plasticity at the transcriptome level.

### Data Accessibility

MIAME compliant data of the microarray experiment have been deposited at Gene Expression Omnibus (http://www.ncbi.nlm.nih.gov/geo/) with accession number GSE52341.

## Supporting Information

Figure S1 **Fold changes of expression levels for Gluthathione S-transferase (Genebank accession nr: EG845649) between temperatures for LIL and for Ependymin precursor (Genebank accession nr: CB502684) between LIL and NOR at 5°C.**
(PDF)

Table S1 **GO-terms identified with enrichment analysis comparing cDNA clones with a population effect against the reference set (8588 analysed cDNA clones).**
(PDF)

Table S2 **GO-terms identified with enrichment analysis comparing cDNA clones with a temperature effect against the reference set (8588 analysed cDNA clones).**
(PDF)

Table S3 **GO-terms identified with enrichment analysis comparing cDNA clones with an interaction effect against the reference set (8588 analysed cDNA clones).**
(PDF)

Table S4 **GO-terms identified with enrichment analysis comparing 90 cDNA clones with an interaction effect against the reference set (8588 analysed cDNA clones).**
(PDF)
