# Supplementary material for: Local Adaptation at the Transcriptome Level in Brown Trout: Evidence from Early Life History Temperature Genomic Reaction Norms
Source: PLoS One. 2014 Jan 14;9(1):e85171. doi: 10.1371/journal.pone.0085171 (PMC3891768; doi:10.1371/journal.pone.0085171)

Figure S1. Fold changes of expression levels for Gluthathione S-transferase (Genebank accession nr: EG845649) between temperatures for LIL and for Ependymin precursor (Genebank accession nr: CB502684) between LIL and NOR at 5°C. Expression levels are given as base 2 logarithms of fold change (± SD). The positive FC indicates that the cDNA clones is up-regulated at 5°C relative to 8°C (Gluthathion S-transferase) and up-regulated in LIL compared to NOR (ependymin precursor).

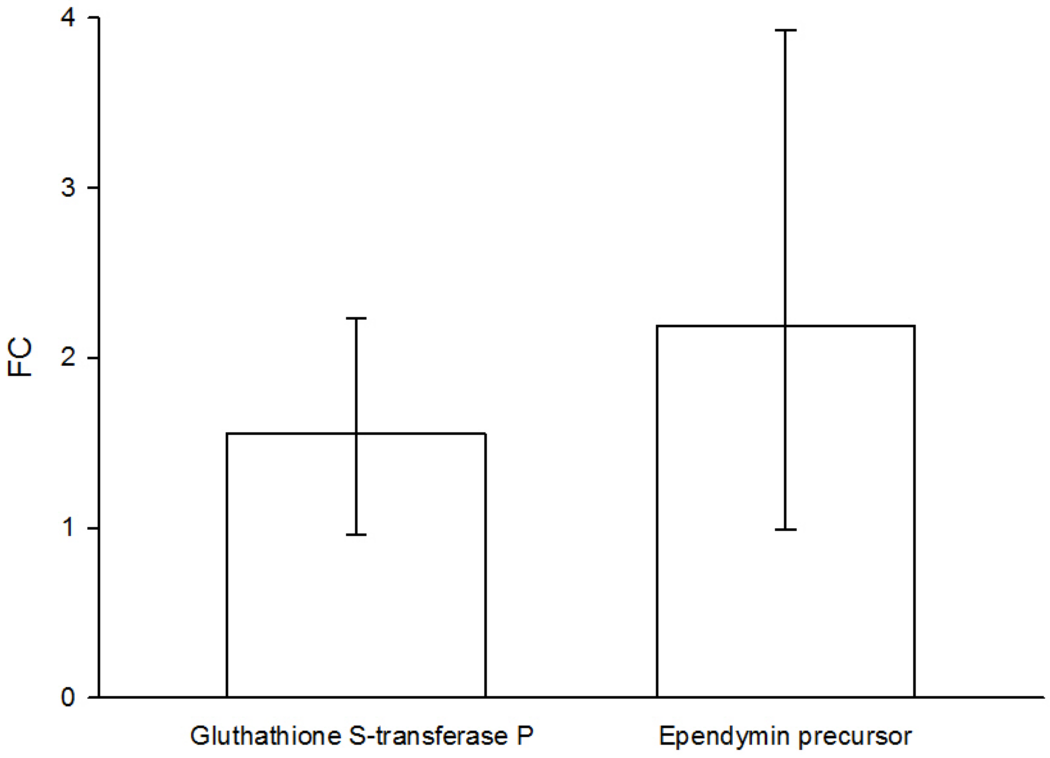

Supplement: Figure S1 — Fold changes of expression levels for Gluthathione S-transferase (Genebank accession nr: EG845649) between temperatures for LIL and for Ependymin precursor (Genebank accession nr: CB502684) between LIL and NOR at 5°C. (PDF) [file pone.0085171.s001.pdf]
