# Supplementary material for: Local Adaptation at the Transcriptome Level in Brown Trout: Evidence from Early Life History Temperature Genomic Reaction Norms
Source: PLoS One. 2014 Jan 14;9(1):e85171. doi: 10.1371/journal.pone.0085171 (PMC3891768; doi:10.1371/journal.pone.0085171)
Supplement: Table S2 — GO-terms identified with enrichment analysis comparing cDNA clones with a temperature effect against the reference set (8588 analysed cDNA clones). (PDF) [file pone.0085171.s003.pdf]

Table S2. GO-terms identified with enrichment analysis comparing cDNA clones with a temperature effect against the reference set (8588 analysed cDNA clones) with BLAST2GO [1,2]. The following information is given: GO-ID, term, p-value after correcting for the False Discovery rate (FDR) [3], number of cDNA clones representing that GO-term with a temperature effect (Test), and among the 8588 cDNA clones (Reference).

GO-ID	Term	FDR corrected p-value	Test	Reference
GO:0006123	mitochondrial electron transport, cytochrome c to oxygen	7E-12	77	40
GO:0022904	respiratory electron transport chain	4E-11	99	69
GO:0042773	ATP synthesis coupled electron transport	1E-10	96	68
GO:0042775	mitochondrial ATP synthesis coupled electron transport	1E-10	96	68
GO:0022900	electron transport chain	1E-09	105	84
GO:0045333	cellular respiration	3E-06	105	106
GO:0015671	oxygen transport	4E-06	39	19
GO:0015669	gas transport	4E-06	39	19
GO:0044260	cellular macromolecule metabolic process	7E-06	387	1108
GO:0055114	oxidation reduction	1E-05	178	232
GO:0015980	energy derivation by oxidation of organic compounds	2E-05	105	113
GO:0044267	cellular protein metabolic process	3E-05	262	792
GO:0006118	electron transport	1E-04	70	67
GO:0022613	ribonucleoprotein complex biogenesis	2E-04	125	426
GO:0042254	ribosome biogenesis	3E-04	122	416
GO:0006091	generation of precursor metabolites and energy	4E-04	173	243
GO:0007586	digestion	4E-04	14	2
GO:0010467	gene expression	5E-04	250	734
GO:0006412	translation	5E-04	163	516
GO:0015992	proton transport	5E-04	119	151
GO:0006818	hydrogen transport	5E-04	119	151
GO:0006812	cation transport	5E-04	157	218
GO:0006119	oxidative phosphorylation	6E-04	117	148
GO:0015672	monovalent inorganic cation transport	6E-04	124	161

GO:0051179	localization	7E-04	329	543
GO:0002474	antigen processing and presentation of peptide antigen via MHC class I	7E-04	17	5
GO:0048002	antigen processing and presentation of peptide antigen	7E-04	17	5
GO:0009059	macromolecule biosynthetic process	9E-04	247	718
GO:0016310	phosphorylation	2E-03	135	185
GO:0006811	ion transport	2E-03	162	234
GO:0006810	transport	2E-03	312	518
GO:0051234	establishment of localization	2E-03	312	520
GO:0006879	cellular iron ion homeostasis	2E-03	30	20
GO:0055072	iron ion homeostasis	2E-03	30	20
GO:0034645	cellular macromolecule biosynthetic process	3E-03	240	688
GO:0006826	iron ion transport	3E-03	27	18
GO:0006793	phosphorus metabolic process	3E-03	144	207
GO:0006796	phosphate metabolic process	3E-03	144	207
GO:0051258	protein polymerization	5E-03	41	37
GO:0044085	cellular component biogenesis	6E-03	192	561
GO:0043170	macromolecule metabolic process	9E-03	491	1259
GO:0007018	microtubule-based movement	1E-02	28	21

References

1. Conesa A, Gotz S, Garcia-Gomez JM, Terol J, Talon M, et al. (2005) Blast2GO: a universal tool for annotation, visualization and analysis in functional genomics research. Bioinformatics 21: 3674-3676.
2. Gotz S, Garcia-Gomez JM, Terol J, Williams TD, Nagaraj SH, et al. (2008) High-throughput functional annotation and data mining with the Blast2GO suite. Nucleic Acids Research 36: 3420-3435.
3. Benjamini Y, Yekutieli D (2001) The control of the false discovery rate in multiple testing under dependency. Annals of Statistics 29: 1165-1188.
