# Supplementary material for: Local Adaptation at the Transcriptome Level in Brown Trout: Evidence from Early Life History Temperature Genomic Reaction Norms
Source: PLoS One. 2014 Jan 14;9(1):e85171. doi: 10.1371/journal.pone.0085171 (PMC3891768; doi:10.1371/journal.pone.0085171)
Supplement: Table S3 — GO-terms identified with enrichment analysis comparing cDNA clones with an interaction effect against the reference set (8588 analysed cDNA clones). (PDF) [file pone.0085171.s004.pdf]

Table S3. GO-terms identified with enrichment analysis comparing cDNA clones with an interaction effect against the reference set (8588 analysed cDNA clones) with BLAST2GO [1,2]. The following information is given: GO-ID, term, p-value after correcting for the False Discovery rate (FDR) [3], number of cDNA clones representing that GO-term with a interaction (Test), and among the 8588 cDNA clones (Reference).

GO-ID	Term	FDR corrected p-value	Test	Reference
GO:0044282	small molecule catabolic process	7E-09	32	114
GO:0016052	carbohydrate catabolic process	7E-09	30	100
GO:0005975	carbohydrate metabolic process	7E-09	38	164
GO:0016051	carbohydrate biosynthetic process	1E-08	27	82
GO:0006096	glycolysis	4E-08	25	76
GO:0006094	gluconeogenesis	8E-08	23	66
GO:0009612	response to mechanical stimulus	8E-08	9	2
GO:0046364	monosaccharide biosynthetic process	8E-08	23	68
GO:0019319	hexose biosynthetic process	8E-08	23	68
GO:0046365	monosaccharide catabolic process	8E-08	25	82
GO:0019320	hexose catabolic process	8E-08	25	82
GO:0006007	glucose catabolic process	8E-08	25	82
GO:0046164	alcohol catabolic process	1E-07	25	84
GO:0044275	cellular carbohydrate catabolic process	1E-07	25	84
GO:0019882	antigen processing and presentation	1E-07	18	39
GO:0006090	pyruvate metabolic process	1E-07	24	78
GO:0046165	alcohol biosynthetic process	2E-07	23	73
GO:0006006	glucose metabolic process	3E-07	26	97
GO:0034637	cellular carbohydrate biosynthetic process	3E-07	23	75
GO:0019318	hexose metabolic process	5E-07	26	102
GO:0005996	monosaccharide metabolic process	8E-07	26	105
GO:0090257	regulation of muscle system process	4E-06	9	7
GO:0006937	regulation of muscle contraction	4E-06	9	7
GO:0007160	cell-matrix adhesion	4E-06	10	11

GO:0031589	cell-substrate adhesion	4E-06	10	11
GO:0045988	negative regulation of striated muscle contraction	4E-06	7	2
GO:0045932	negative regulation of muscle contraction	4E-06	7	2
GO:0031448	positive regulation of fast-twitch skeletal muscle fiber contraction	4E-06	7	2
GO:0003009	skeletal muscle contraction	4E-06	7	2
GO:0003010	voluntary skeletal muscle contraction	4E-06	7	2
GO:0014721	twitch skeletal muscle contraction	4E-06	7	2
GO:0014819	regulation of skeletal muscle contraction	4E-06	7	2
GO:0045933	positive regulation of muscle contraction	4E-06	7	2
GO:0014724	regulation of twitch skeletal muscle contraction	4E-06	7	2
GO:0045989	positive regulation of striated muscle contraction	4E-06	7	2
GO:0031446	regulation of fast-twitch skeletal muscle fiber contraction	4E-06	7	2
GO:0031443	fast-twitch skeletal muscle fiber contraction	4E-06	7	2
GO:0050879	multicellular organismal movement	9E-06	7	3
GO:0050881	musculoskeletal movement	9E-06	7	3
GO:0006942	regulation of striated muscle contraction	9E-06	7	3
GO:0006200	ATP catabolic process	9E-06	7	3
GO:0009261	ribonucleotide catabolic process	9E-06	7	3
GO:0009143	nucleoside triphosphate catabolic process	9E-06	7	3
GO:0006195	purine nucleotide catabolic process	9E-06	7	3
GO:0009203	ribonucleoside triphosphate catabolic process	9E-06	7	3
GO:0009154	purine ribonucleotide catabolic process	9E-06	7	3
GO:0009146	purine nucleoside triphosphate catabolic process	9E-06	7	3
GO:0009207	purine ribonucleoside triphosphate catabolic process	9E-06	7	3
GO:0006066	alcohol metabolic process	1E-05	26	129
GO:0006955	immune response	1E-05	18	63
GO:0032787	monocarboxylic acid metabolic process	1E-05	24	112
GO:0044262	cellular carbohydrate metabolic process	1E-05	26	130
GO:0071554	cell wall organization or biogenesis	2E-05	10	15

GO:0044036	cell wall macromolecule metabolic process	2E-05	10	15
GO:0030001	metal ion transport	3E-05	23	108
GO:0034656	nucleobase, nucleoside and nucleotide catabolic process	4E-05	7	5
GO:0034655	nucleobase, nucleoside, nucleotide and nucleic acid catabolic process	4E-05	7	5
GO:0009166	nucleotide catabolic process	4E-05	7	5
GO:0015674	di-, tri-valent inorganic cation transport	5E-05	16	55
GO:0046700	heterocycle catabolic process	9E-05	7	6
GO:0055066	di-, tri-valent inorganic cation homeostasis	1E-04	16	59
GO:0030005	cellular di-, tri-valent inorganic cation homeostasis	1E-04	16	59
GO:0055074	calcium ion homeostasis	1E-04	9	15
GO:0055065	metal ion homeostasis	1E-04	9	15
GO:0006875	cellular metal ion homeostasis	1E-04	9	15
GO:0006874	cellular calcium ion homeostasis	1E-04	9	15
GO:0009056	catabolic process	1E-04	43	332
GO:0043170	macromolecule metabolic process	2E-04	62	1688
GO:0044270	cellular nitrogen compound catabolic process	2E-04	7	7
GO:0055080	cation homeostasis	2E-04	16	64
GO:0030003	cellular cation homeostasis	2E-04	16	64
GO:0070838	divalent metal ion transport	2E-04	9	17
GO:0006816	calcium ion transport	2E-04	9	17
GO:0044283	small molecule biosynthetic process	3E-04	37	275
GO:0042180	cellular ketone metabolic process	4E-04	37	279
GO:0002376	immune system process	4E-04	18	85
GO:0006873	cellular ion homeostasis	4E-04	16	68
GO:0051241	negative regulation of multicellular organismal process	4E-04	7	9
GO:0006941	striated muscle contraction	4E-04	7	9
GO:0006936	muscle contraction	4E-04	10	25
GO:0003012	muscle system process	4E-04	10	25
GO:0055082	cellular chemical homeostasis	4E-04	16	69

GO:0050801	ion homeostasis	4E-04	16	69
GO:0051240	positive regulation of multicellular organismal process	6E-04	7	10
GO:0048878	chemical homeostasis	7E-04	16	72
GO:0043436	oxoacid metabolic process	7E-04	33	245
GO:0006082	organic acid metabolic process	7E-04	33	245
GO:0019752	carboxylic acid metabolic process	7E-04	33	245
GO:0009628	response to abiotic stimulus	7E-04	10	27
GO:0008652	cellular amino acid biosynthetic process	3E-03	10	34
GO:0044057	regulation of system process	4E-03	9	28
GO:0016998	cell wall macromolecule catabolic process	4E-03	6	10
GO:0006022	aminoglycan metabolic process	5E-03	8	22
GO:0006542	glutamine biosynthetic process	8E-03	4	3
GO:0009309	amine biosynthetic process	9E-03	10	40

References

1. Conesa A, Gotz S, Garcia-Gomez JM, Terol J, Talon M, et al. (2005) Blast2GO: a universal tool for annotation, visualization and analysis in functional genomics research. Bioinformatics 21: 3674-3676.
2. Gotz S, Garcia-Gomez JM, Terol J, Williams TD, Nagaraj SH, et al. (2008) High-throughput functional annotation and data mining with the Blast2GO suite. Nucleic Acids Research 36: 3420-3435.
3. Benjamini Y, Yekutieli D (2001) The control of the false discovery rate in multiple testing under dependency. Annals of Statistics 29: 1165-1188.
