# Supplementary material for: Local Adaptation at the Transcriptome Level in Brown Trout: Evidence from Early Life History Temperature Genomic Reaction Norms
Source: PLoS One. 2014 Jan 14;9(1):e85171. doi: 10.1371/journal.pone.0085171 (PMC3891768; doi:10.1371/journal.pone.0085171)
Supplement: Table S4 — GO-terms identified with enrichment analysis comparing 90 cDNA clones with an interaction effect against the reference set (8588 analysed cDNA clones). (PDF) [file pone.0085171.s005.pdf]

Table S4. GO-terms identified with enrichment analysis comparing 90 cDNA clones with an interaction effect against the reference set (8588 analysed cDNA clones) with BLAST2GO [1,2]. The following information is given: GO-ID, term, p-value after correcting for the False Discovery rate (FDR) [3], number of cDNA clones representing that GO-term among the 90 cDNA clones with an interaction (Test), and among the 8588 cDNA clones (Reference).

GO-ID	Term	FDR corrected p-value	Test	Reference
GO:0007160	cell-matrix adhesion	3E-09	9	12
GO:0031589	cell-substrate adhesion	3E-09	9	12
GO:0019882	antigen processing and presentation	2E-05	9	48
GO:0007155	cell adhesion	2E-05	9	51
GO:0022610	biological adhesion	2E-05	9	51
GO:0006955	immune response	3E-04	9	72
GO:0002376	immune system process	2E-03	9	94
GO:0006886	intracellular protein transport	4E-03	8	84
GO:0070727	cellular macromolecule localization	5E-03	8	88
GO:0034613	cellular protein localization	5E-03	8	88

References

1. Conesa A, Gotz S, Garcia-Gomez JM, Terol J, Talon M, et al. (2005) Blast2GO: a universal tool for annotation, visualization and analysis in functional genomics research. Bioinformatics 21: 3674-3676.
2. Gotz S, Garcia-Gomez JM, Terol J, Williams TD, Nagaraj SH, et al. (2008) High-throughput functional annotation and data mining with the Blast2GO suite. Nucleic Acids Research 36: 3420-3435.
3. Benjamini Y, Yekutieli D (2001) The control of the false discovery rate in multiple testing under dependency. Annals of Statistics 29: 1165-1188.
